# Type I interferon (IFN)-inducible Absent in Melanoma 2 proteins in neuroinflammation: implications for Alzheimer’s disease

**DOI:** 10.1186/s12974-019-1639-5

**Published:** 2019-11-26

**Authors:** Divaker Choubey

**Affiliations:** Department of Environmental Health, University of Cincinnati, 160 Panzeca Way, P. O. Box 670056, Cincinnati, OH 45267 USA

**Keywords:** Type I IFNs, Alzheimer’s disease, Neuroinflammation, AIM2 proteins

## Abstract

Cumulative evidence indicates that activation of innate immune responses in the central nervous system (CNS) induces the expression of type 1 interferons (T1 IFNs), a family of cytokines. The T1 IFNs (IFN-α/β), through activation of the JAK/STAT-signaling in microglia, astrocytes, and neurons, induce the expression of IFN-inducible proteins, which mediate the pro- and anti-inflammatory functions of IFNs. Accordingly, T1 IFN-inducible Absent in Melanoma 2 proteins (murine Aim2 and human AIM2) negatively regulate the expression of TI IFNs and, upon sensing higher levels of cytosolic DNA, assemble the Aim2/AIM2 inflammasome, resulting in activation of caspase-1, pyroptosis, and the secretion of pro-inflammatory cytokines (e.g., IL-1β and IL-18). Of interest, studies have indicated a role for the Aim2/AIM2 proteins in neuroinflammation and neurodegenerative diseases, including Alzheimer’s disease (AD). The ability of Aim2/AIM2 proteins to exert pro- and anti-inflammatory effects in CNS may depend upon age, sex hormones, cell-types, and the expression of species-specific negative regulators of the Aim2/AIM2 inflammasome. Therefore, we discuss the role of Aim2/AIM2 proteins in the development of AD. An improved understanding of the role of Absent in Melanoma 2 proteins in AD could identify new approaches to treat patients.

## Introduction

In the central nervous system (CNS), most cell-types, including microglia and astrocytes, can produce type 1 interferons (T1 IFNs) [1, 2], a family of cytokines [1]. The T1 IFNs signal via the heterodimeric IFN-α/β receptor (IFNAR). Binding of T1 IFNs with the receptor activates the JAK/STAT-signaling pathway leading to transcriptional activation of the IFN-stimulated genes (ISGs) [1]. These genes encode for the proteins that mediate the pro- and anti-inflammatory functions of the T1 IFNs [2, 3]. T1 IFN response in the CNS may arise due to certain bacterial or viral infections [4, 5]. Alternatively, traumatic brain injury (TBI) and neurodegeneration can also activate T1 IFN response [6–11]. Accordingly, a recent study reported expression of T1 IFN-inducible Absent in Melanoma 2 (Aim2) protein in the murine astrocytes and microglia [12].

Depending on the neurodegenerative disease state, T1 IFNs can be protective or deleterious [2]. For example, in multiple sclerosis (MS), a neuroinflammatory diseases, the T1 IFNs are thought to exert an anti-inflammatory effect through induction of the anti-inflammatory cytokine IL10 [13] and suppression of pro-inflammatory cytokine IL-1β production [14]. Consequently, the IFN-β is a first-line therapy for certain MS patients. The therapy limits infiltration of lymphocytes into the brain and decreases relapse rate in MS patients [2, 15]. However, a transgenic overexpression of the IFN-α gene in the brains of mice resulted in neuroinflammation and the development of a neurodegenerative disease [16, 17]. Correspondingly, the IFN-β levels are increased with age in individuals and an administration of anti-IFNAR1 antibodies in individuals inhibited the age-dependent cognitive decline [18].

Generation of the IFNAR1-null [19] or IFN-β^−/−^ mice [20] improved our overall understanding with respect to the role of T1 IFNs and the T1 IFN-signaling in neurodegenerative diseases. Surprisingly, the mice that were deficient in the *Ifnb* gene (encoding for IFN-β) exhibited a loss of the dopaminergic neurons, development of Lewy bodies and Parkinson’s-like disease [20]. Of interest, the animal models of AD exhibited an increase in the expression of T1 IFNs and activation of the T1 IFN response in the CNS [21, 22]. Accordingly, the APP_SWE_ /PS1_ΔE9_ AD mice that were deficient in the IFN-α receptor gene (*Ifnar1*) exhibited a reduced pathology and an altered microglial phenotype [11]. Similarly, a study using a mouse model of Alzheimer’s disease (5XFAD) indicated an inflammasome-independent role of T1 IFN-inducible Aim2 protein in the suppression of pro-inflammatory cytokines in the *Aim*^*−*/*−*^.(B6.Sv129); 5XFAD mice [23].

Type 1 (T1) interferonopathies are characterized by the constitutive production of T1 IFNs and activation of T1 IFN response (increase in the expression of ISGs) in cells [24–26]. The T1 interferonopathies usually involve mutation in genes that regulate the T1 IFN response [25, 26]. Interestingly, certain types of T1 interferonopathies, including the Aicardi-Goutieres syndrome (AGS), are associated with activation of microglia in the CNS, resulting in chronic neuroinflammation [26, 27]. Further, chronic neuroinflammation is associated with many aging-associated neurodegenerative diseases (including Alzheimer’s disease, Parkinson’s disease, multiple sclerosis, and ataxia telangiectasia) in individuals and animal models of the diseases [28–30].

Accumulating evidence indicates that an inflammasome activation in glial and neuronal cells modulates neuroinflammation [31, 32]. The inflammasome is a cytosolic protein complex that contains multiple copies of a danger-sensing receptor (e.g., Aim2), pro-caspase-1, and an adaptor protein ASC (apoptotic speck containing protein with a CARD) [33]. Activation of an inflammasome in glial and neuronal cells results in caspase-1 activation, pyroptotic cell death, and the release of pro-inflammatory cytokines, such as IL-1β and IL-18 [32, 34]. Activation of inflammasomes plays a pathogenic role in neurologic diseases such as multiple sclerosis, traumatic brain injury, and Alzheimer’s disease [31, 32, 35].

Development of aging-associated Alzheimer’s disease (AD) is characterized by synaptic loss and neuronal death, which results in cognitive decline, dementia, and loss of motor functions with time [36–38]. Deposition of extracellular beta-amyloid (Aβ) plaques and neurofibrillary tangles of the hyperphosphorylated microtubule-binding protein tau in the CNS is thought to play a role in the development and progression of aging-associated AD [36, 38]. However, it remains an open question whether an increase in the production of pro-inflammatory (such as IL-6 and IL-1β) [39] that are produced by activation of an inflammasome in the CNS or anti-inflammatory (such as IL-10) [40, 41] cytokines contributes to the main defects in the clearance of the Aβ plaques in AD patients.

Cumulative evidence indicates a role for the T1 IFN-signaling in the development and progression of AD [11, 21–23, 42]. However, the role of IFN-inducible proteins, which mediate the pro- and anti-inflammatory activities of T1 IFNs in the CNS, remains unknown. Interestingly, studies using animal models have indicated a role for T1 IFN-inducible Aim2 protein in neuroinflammation and neurodegenerative diseases, including AD [12, 23, 43–48]. Because the ability of Aim2/AIM2 proteins to exert pro- and anti-inflammatory effects in the CNS may depend upon the genetic background, age, sex hormones, cell-type, and species-specific expression of the negative regulators of the Aim2/AIM2 inflammasome [49–52], here we discuss the role of these proteins in the development of neurodegenerative diseases.

## Interferon-inducible Absent in Melanoma 2 proteins

The T1 IFN-inducible PYHIN-protein family includes the structurally related Aim2/AIM2 proteins that are encoded by the *AIM2*-like receptor (ALR)-family genes (*ALR* genes) [53–55]. The family includes the murine genes (including the *Aim2* and *Ifi202*) and human genes (including the *AIM2*, *IFI16*, and *POP3*). Notably, the murine Aim2 and human AIM2 protein sequences are conserved between the mouse and humans. Most proteins in the family contain the N-terminal Pyrin signaling domain (PYD) and the C-terminal DNA-binding HIN domain [53]. The murine-specific p202 [53] and human-specific IFI16-β [56] proteins lack the PYD-signaling domain. Further, the human-specific POP3 protein lacks the DNA-binding HIN domain. The HIN domain can bind double-stranded DNA in sequence-independent manner [53]. Interestingly, the p202 protein through the HIN domain interacted with the Aim2 protein and inhibited the activity of the Aim2 inflammasome [57]. Similarly, the T1 IFN-induced levels of the IFI16-β and POP3 protein inhibited the activity of the AIM2 inflammasome [56, 58]. Basal (and T1 IFN-induced) expression of Aim2 and p202 proteins may depend on the mouse strain and sex (see below) [49, 51, 52]. Further, basal expression of the Aim2/AIM2 proteins is detectable in glial cells and astrocytes [12].

Bone marrow-derived macrophages from *Aim*^*−/−*^ female mice on the mixed (B6.Sv129) [50] or pure C57BL/6 (B6) [51] genetic background expressed higher basal levels of the IFN-β and activated the T1 IFN response. Further, *Aim2-*deficient mice on mixed genetic background exhibited inflammation and adipogenesis in white adipose tissue with age, leading to obesity and insulin resistance [59]. Notably, *Aim2-*deficiency in the B6.Sv129 and B6 mice resulted in an increase in the expression of the IFN-inducible p202 protein [50, 51, 59]. Because the basal expression of the p202 protein (a negative regulator of the Aim2 inflammasome) is much higher in the B6.Sv129 mouse strain than the B6 strain [60], studies using the *Aim2*^*−*/*−*^.(B6.Sv129) mice are not very informative with respect to the precise role of the Aim2 protein in the development of neuroinflammation and neurodegenerative diseases.

## Age-dependent expression of *Aim2/AIM2* genes

Analysis of the *AIM2* gene expression in peripheral blood mononuclear cells (PBMCs) from vascular patients (*n* = 77, age 22–82 years) revealed a significant positive association with age [61]. Notably, the analysis did not find a difference in *AIM2* expression between patients with advanced atherosclerosis and other vascular diseases. Similarly, human normal lung fibroblasts (WI-38) in culture, upon aging, exhibited a measurable increase in the levels of the AIM2 protein [62]. However, a study [63], which analyzed *AIM2* gene expression in PBMCs from healthy young (*n* = 16; age 20–39 years) and elderly (*n* = 18; 60–84 years) individuals without any treatment or after in vitro stimulation of cells with poly(dA:dT), an AIM2 ligand, noted that the stimulation of cells from elderly individuals resulted in reduced expression of the *AIM2* gene and pro-inflammatory cytokines than the young donors [63]. Because development of certain neurodegenerative diseases, such as AD, is associated with aging and vascular dysfunction [64], it remains to be seen whether the expression of *AIM2* gene and the functions of the AIM2 protein in the CNS decrease with the age.

## A male bias in the expression of *Aim2*/*AIM2* genes

In purified splenic B cells (B220^+^) and total sleenocytes from the B6, New Zealand black (NZB) and B6.*Nba2* congenic males, as compared with age-matched females, the basal levels of the Aim2 mRNA and protein were significantly higher [49]. Further, treatment of the murine WT276 breast cancer cell line, which expresses the androgen receptor (AR), with dihydroxy-testosterone (DHT) measurably increased the steady-state levels of the *Aim2* mRNA and protein [49]. Similarly, expression of the *AIM2* gene in PBMCs from males (*n* = 62) was higher than females (*n* = 38) [61]. Additionally, *AIM2* mRNA levels in naïve macrophages were higher in SLE men (*n* = 6) than women (*n* = 9) [65]. Considering the above observations, it is conceivable that the basal expression of the *Aim2* and *AIM2* genes in microglia and astrocytes is regulated by the sex hormones in a cell-type dependent manner. Further, expression of the p202 protein [66] and IFI16 protein [67], the negative regulators of the Aim2 and AIM2 inflammasome, is regulated by the sex hormones. Because epidemiological studies suggest that lower androgen levels in elderly men are a risk factor to develop AD [68], studies are needed to investigate whether age-dependent decrease in the androgen levels in men is associated with reduced levels of the AIM2 protein and its functions in the CNS. Additionally, whether a reduced expression of AIM2 protein in women is associated with a female bias in the development of AD.

## Cytosolic DNA sensing: Aim2/AIM2 proteins in innate immune responses

**Fig. 1:**
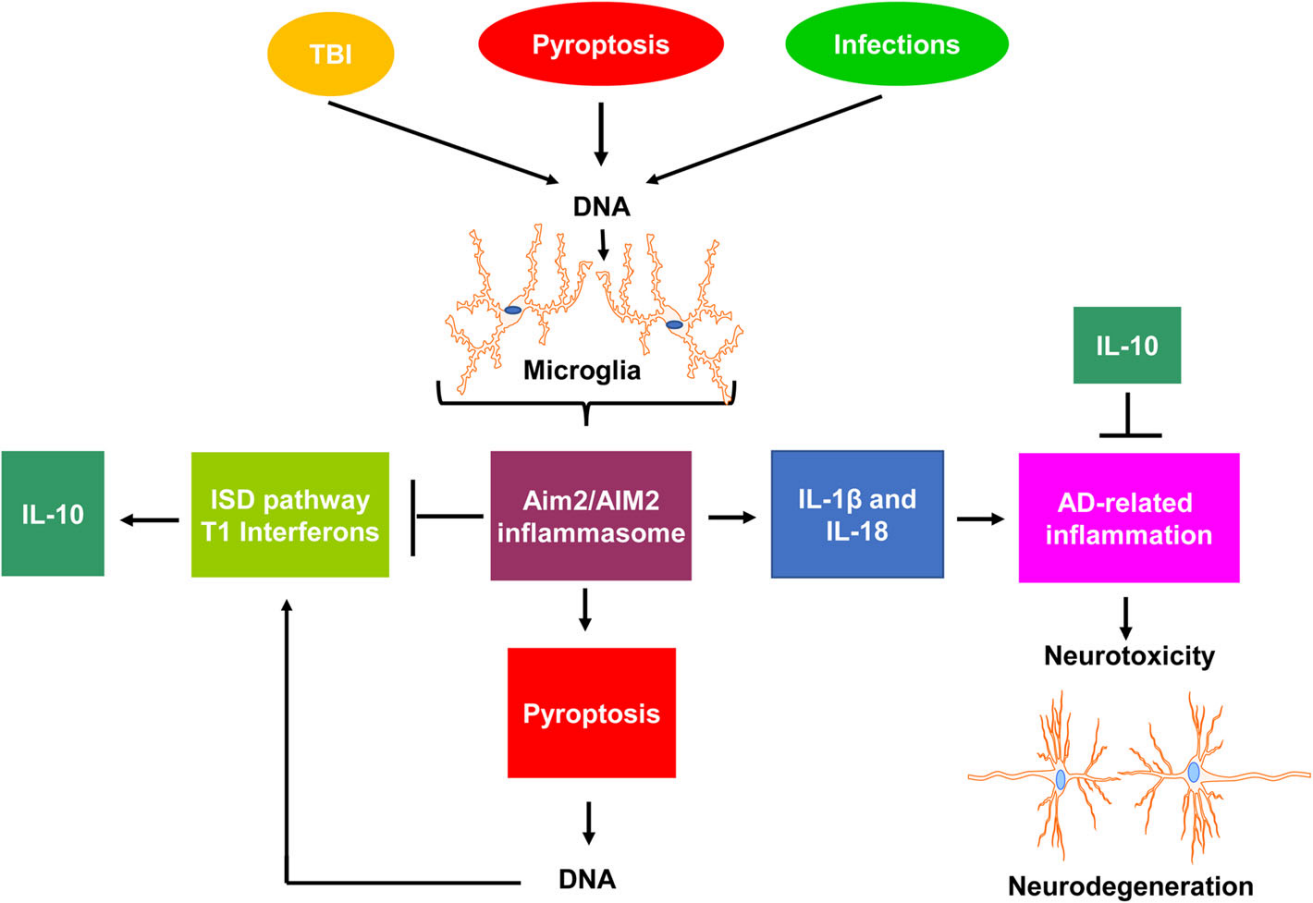
Aim2 protein-mediated modulation of AD-related inflammation. Mouse microglia express detectable basal levels of Aim2 protein. Detection of low levels of cytosolic DNA, released in the brain due to traumatic brain injury (TBI), pyroptosis (caused by activation of inflammasomes), and brain infections, by cGAS activates the IFN-stimulatory DNA (ISD) pathway and the T1 IFN response, resulting in upregulation of the *Aim2* gene expression and anti-inflammatory cytokine IL-10. The IL-10 suppresses neuroinflammation. Detection of increased levels of cytosolic DNA by macrophages/microglia activates the Aim2 inflammasome, resulting in the production of pro-inflammatory cytokines (IL-1β and IL-18) and cell death by pyroptosis. The activation of the Aim2 inflammasome and pyroptosis contributes to AD-related inflammation and associated neurotoxicity, resulting in neurodegeneration. Cell death by pyroptosis inhibits the ISD pathway and the production of IL-10

Lower levels of cytosolic DNA are sensed by cyclic GMP-AMP synthase (cGAS) in macrophages/microglia [51, 52, 69]. Upon sensing the cytosolic DNA, macrophages activate the STING-dependent IFN-stimulatory DNA pathway (ISD; also referred to as the cGAS-STING-TBK1-IRF3 pathway) for the IFN-β expression and activation of the T1 IFN response [70, 71], which upregulates the expression of *Aim2*/*AIM2* genes [49, 61] and interleukin-10 (IL-10) [14]. However, higher levels of the cytosolic DNA are sensed by T1 IFN-inducible murine Aim2 and human AIM2 proteins in macrophages and the sensing activates the Aim2/AIM2 inflammasome and caspase-1 [69]. The activated caspase-1 proteolytically cleaves pro-IL-1β (p31), pro-IL-18 (p24), and gasdermin D, which leads to pyroptosis, a highly inflammatory cell death, and inflammation [72]. Notably, the activated gasdermin D in macrophages also limited activation of the T1 IFN responses [73]. These observations are consistent with a role for activation of the ISD pathway by lower levels of the cytosolic DNA to potentiate the activation of the Aim2/AIM2 inflammasome (through stimulation of the *Aim2*/*AIM2* gene expression) in macrophages/microglia. In turn, activation of the Aim2/AIM2 inflammasome by higher levels of the cytosolic DNA in macrophages negatively regulates the ISD pathway to suppress T1 IFN response. Therefore, a mutual regulation between activation of ISD pathway and inflammasome pathway in the CNS may be critical to maintain a homeostasis (Fig. 1).

## The negative regulation of type I IFN response by Aim2/AIM2 proteins

**Fig. 2:**
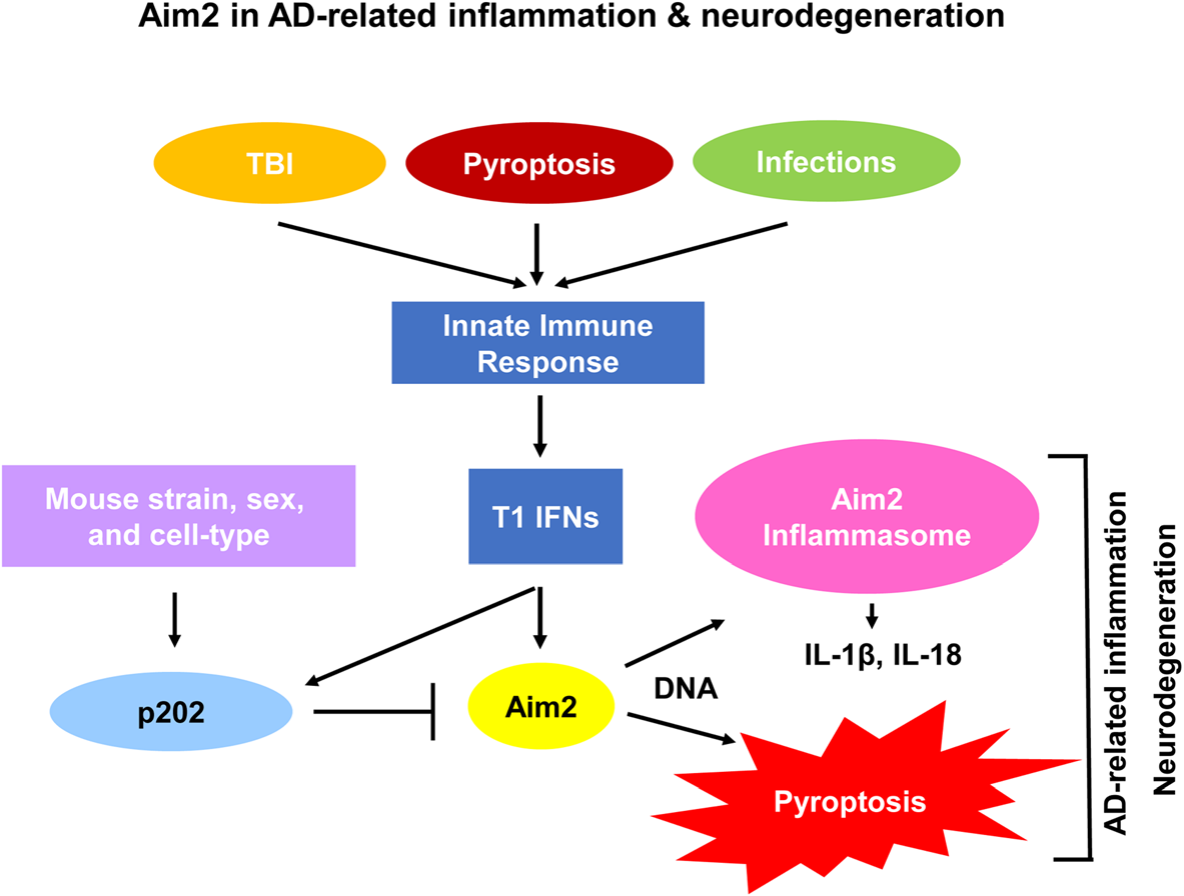
Aim2 and p202 proteins in modulation of AD-related inflammation and neurodegeneration. TBI, pyroptosis, and brain infections activate innate immune responses in microglia through stimulation of TLRs and ISD pathway, resulting in the T1 IFN response and upregulation of the *Aim2* and *Ifi202* gene expression. A higher Aim2-to-p202 protein ratio in macrophages/microglia, which depends upon the genetic background of the mice, sex, and cell-type, is predicted to result in activation of the Aim2 inflammasome and pyroptosis, resulting in AD-related inflammation and neurodegeneration. However, a higher p202-to-Aim2 protein ratio in microglia is predicted to suppress AD-related inflammation and maintain homeostasis in the CNS

Immune cells (spleenocytes, macrophages, and dendritic cells) from *Aim2-*deficient male or female mice expressed higher basal levels of the IFN-β mRNA and activated the T1 IFN response as compared with the age- and sex-matched wild-type B6 mice [50, 51]. Further, macrophages and DCs from the B6 female mice, when stimulated in vitro with DNA, activated the Aim2 inflammasome activity and inhibited activation of ISD pathway by caspase-1-mediated pyroptotic cell death [73]. However, in vitro stimulation of macrophages from the NZB female mice, which express higher basal levels of the p202 protein than the B6 mice [74], resulted in an inhibition of Aim2 inflammasome activity as compared with sex-matched B6 mice [57]. Additionally, Aim2 protein sequestered the T1 IFN-inducible IFI205 protein and inhibited the IFI205-mediated induction of IFN-β through activation of ISD pathway [75]. Further, Aim2 protein also potentiated the Trex1-mediated suppression of T1 IFN response in macrophages through mechanisms dependent upon the cGas and Sting proteins [75]. Together, these observations suggest that Aim2 protein negatively regulates the T1 IFN response in inflammasome-dependent and -independent manner. Further, the Aim2 inflammasome-dependent pro-inflammatory response depends on the genetic background of the mice and their sex (Fig. 2).

Constitutive expression of the POP3 protein, a negative regulator of the AIM2 inflammasome [58], in murine macrophages “primed” macrophages, resulting in a significant increase in the production of the IFN-β upon sensing of the cytosolic DNA [58]. Accordingly, a knockdown of the AIM2 protein expression in human normal lung fibroblasts (WI-38) activated the T1 IFN response and upregulated the expression of the IFN-inducible IFI16 protein [62], an activator of the ISD pathway [76]. Further, an increase in the expression of IFI16 protein [77] or its IFI16-β isoform [56], which contains two HIN domains, sequestered the cytoplasmic DNA and inhibited activation of the AIM2 inflammasome [56, 77]. Considering the above observations, further work is needed to investigate the role of AIM2 protein in the negative regulation of T1 IFN response.

## Aim2 protein expression in the CNS and neuroinflammation

An extensive expression analysis of T1 IFN-inducible cytosolic DNA sensors in highly purified primary astrocytes and microglia indicated that both cell-types express mRNAs encoding for the PYHIN-family proteins, including the Aim2 protein [12]. Treatment of microglia with IFN-β upregulated the expression of certain cytosolic DNA sensors, including the *Aim2* gene. Interestingly, in a murine model of chronic neurodegeneration, the expression of *Aim2* gene was upregulated in vivo in a T1 IFN-dependent fashion [12]. Further, acute CNS infection by *S. aureus* in mice resulted in the production of IL-1β and other inflammatory chemokines and cytokines (including IL-6 and CXCL1) [44]. Together, these observations support the idea that upregulation of the *Aim2* gene expression in the CNS by T1 IFNs or by an infection by bacteria may contribute to neuroinflammation.

Cultured cortical and hippocampal mouse neurons expressed higher basal levels of *Aim2* mRNA and treatment of neurons with synthetic double-stranded DNA induced IL-1β secretion in *Aim2*-dependent manner [46]. Interestingly, activation of the Aim2 inflammasome in neurons and IL-1β-induced signaling downregulated dendritic cell growth but enhanced axon extension. Further, a knockdown or knockout of the *Aim2* gene expression in neurons indicated that Aim2 protein acted in cell-autonomous manner to regulate the neuronal morphology. Notably, the behavioral analyses of the Aim2^−/−^.(B6.Sv129) mice revealed that the mice exhibited lower locomotor activity, increased anxious behaviors, and a reduced auditory fear memory [46]. However, it remains unclear whether the phenotype exhibited by the Aim2^−/−^.(B6.Sv129) mice is due to activation of the T1 IFN response, which increases the expression of the p202 protein.

## Aim2 protein in traumatic brain and spinal cord injuries

Using a controlled cortical impact (CCI) mouse model for traumatic brain injury (TBI) and manipulating the extent of pyroptosis in blood-brain barrier (BBB) cells, one study noted that TBI resulted in Aim2 inflammasome-mediated pyroptosis in brain microvascular endothelial cells (BMVECs) within injured cerebral cortex region [47]. Further, treatment with an inhibitor of caspase-1 (Ac-YVAD-cmk), which inhibited pyroptosis in the BMVECs and the release of pro-inflammatory cytokines (IL-1β and IL-18), decreased the TBI-induced blood-brain barrier leakage, brain edema, loss of tight junction proteins, and the inflammatory response in injured BMVECs. Correspondingly, the treatment also improved the neurological outcome of CCI in the mice. Similarly, using a rodent model of stroke, a study showed that Aim2 inflammasome activation contributes to brain injury [45]. Together, these observations may suggest a role for the Aim2 inflammasome activation in TBI and stroke-associated neuroinflammation.

In a rat model of T9 spinal cord contusive injury (SCI), levels of the Aim2 protein were measurably higher than sham-operated rats after 1 h, 6 h, and 1 day of the injury [48]. Further, the basal expression of the *Aim2* gene was detectable in neurons, astrocytes, oligodendrocytes, and microglia in the sham-operated spinal cord. In rats with SCI, Aim2 protein was also detectable in leukocytes and activated microglia/macrophages (CD68^+^ cells) in the spinal cord. These observations are consistent with the possibility that Aim2 protein is expressed in the normal spinal cord, and after SCI, expression of the *Aim2* gene increases in activated astrocytes and microglia due to the secretion of certain cytokines by infiltrated leukocytes.

## Aim2 in Alzheimer’s disease

Using the wild-type B6, Aim2^−/−^.(B6.Sv129), 5XFAD, and Aim2^−/−^.(B6.Sv129); 5XFAD mice, a study noted that *Aim2* gene deficiency in the Aim2^−/−^.(B6.Sv129); 5XFAD mice mitigated the Aβ deposition in the cerebral cortex and hippocampus region as compared with the parental 5XFAD mice [23]. Further, activation of microglia was decreased in the brains of *Aim2*^−/−^.(B6.Sv129); 5XFAD mice as compared with age-matched 5XFAD mice. Surprisingly, the *Aim2*^−/−^.(B6.Sv129) mice did not exhibit an improved memory and reduced anxiety as compared with 5XFAD mice in behavioral tests [23]. Compared with 5XFAD mice, IL-1 expression did not decrease in the *Aim2*^−/−^.(B6.Sv129); 5XFAD mice, thus suggesting a role for other inflammasomes in the observed phenotype. Of interest, IL-6 and IL-18 expression was higher in brains from the *Aim2*^−/−^(B6. Sv129); 5XFAD mice than the 5XFAD mice [23].

In another study [78], the APP/PS1 male mice (synonymous to the 5XFAD mice, age 6 months) exhibited increased levels of the Aim2 protein in the hippocampus (in particular, in the CA1 neurons). Further, *Aim2* gene deletion in APP/PS1 mice increased spatial memory in behavioral tests, facilitated the long-term potentiation in hippocampal slices, altered the structure of dendrites, and increased the dendritic spine densities. Notably, a transcriptional analysis revealed that *Aim2* gene deletion in the APP/PS1 mice altered the expression levels of certain proteins, including Pten, Homer1, and Ppp2r3a, and increased activation of the AKT protein kinase [78]. These observations are consistent with a role of the Aim2 inflammasome activation in neuroinflammation.

In *Aim2*^−/−^.(B6.Sv129); 5XFAD AD mouse model, the lack of Aim2 protein function reduced Aβ deposition in the brain [23]. Considering that *Aim2* gene deficiency in the mice is predicted to increase the expression of T1 IFN-β and activate a T1 IFN response [50, 51] and activation of the T1 IFN response in microglia increased *Trem2* mRNA levels [79], which encodes for the TREM2 receptor that regulates microglial functions (including phagocytosis of Aβ deposits) [80], it is conceivable that the *Aim2* gene deficiency reduced the Aβ deposits in the *Aim2*^−/−^.(B6.Sv129); 5XFAD AD mouse model through upregulation of the *Trem2* gene expression. Therefore, it is important to determine the role of Aim2 protein and its negative regulators in the regulation of Aβ deposition.

In *Aim2*^−/−^.(B6.Sv129); 5XFAD mice, *Aim2* gene deficiency increased the levels of certain pro-inflammatory cytokines (including IL-6) in the brain and had limited effect on the cognitive behavior of the mice. As noted above, *Aim2* gene deficiency in the B6.Sv129 or B6 mice activated a T1 IFN response, resulting in an increase in the expression of p202 protein (encoded by the *Ifi202* gene) [50, 51]. The p202 protein is a transcriptional modulator, which can modulate the activities of the E2Fs, p53, NF-κB, and MyoD transcription factors [54]. Therefore, it is not surprising that the APP/PS1 mice deficient in the *Aim2* gene exhibited the transcriptional changes in certain genes [78]

Interestingly, *Aim2* gene deficiency in the B6.Sv129 mice resulted in inflammation in white adipose tissue and associated with an increase in the expression of the *Ifi202* gene [59]. Further, a knockdown of *Ifi202* gene expression in stromal vascular fraction (SVF) inhibited adipogenesis and inflammation [59]. Additionally, bone marrow macrophages from the *Aim2-*deficient B6.Sv129 mice, upon challenge with LPS, expressed significantly higher levels of IL-6 mRNA [59]. Together, these observations support the hypothesis that Aim2 protein expression in 5XFAD or APP/PS1 AD mice suppresses neuroinflammation in part by suppressing the expression of certain T1 IFN-inducible proteins, including the p202 protein.

## Regulation of the ATM protein kinase by Aim2/AIM2 proteins and neurodegeneration

**Fig. 3:**
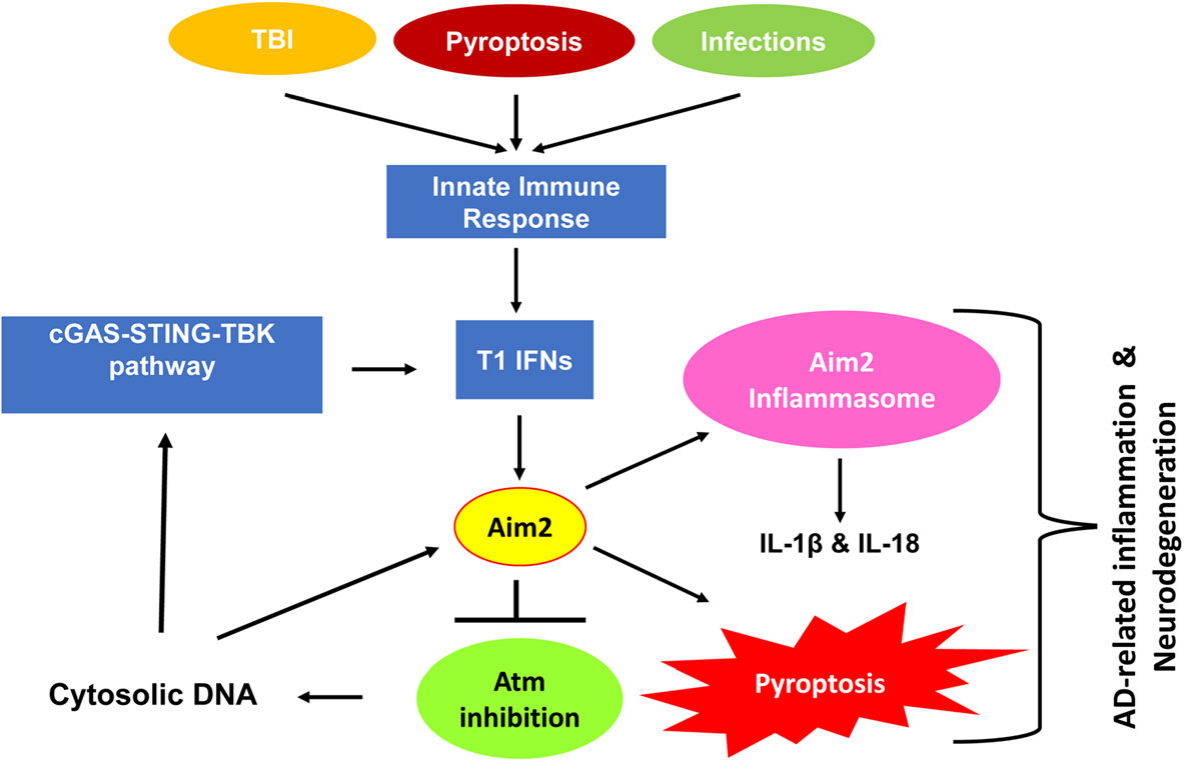
The Atm-Aim2 axis in neuroinflammation and neurodegeneration. A knockdown of the AIM2 protein expression in human normal lung fibroblasts or a deficiency of the *Aim2* gene in the murine bone marrow-derived macrophages activated ATM protein kinase (a member of the PI-3-kinase/DNA-PK family, which can bind to Aim2 protein), thus suggesting a role for the Aim/AIM2 protein in inhibition of the ATM protein kinase activity. A dysfunction of ATM protein kinase in microglia accumulated DNA fragments in the cytoplasm, resulting in activation of the ISD pathway and T1 IFN response (the accumulation of cytosolic DNA did not result in activation of an inflammasome and pyroptosis). The T1 IFN response, which upregulates the expression of Aim2 protein, is predicted to “prime” microglia for activation of the Aim2/AIM2 inflammasome upon sensing higher levels of cytosolic DNA (due to TBI, pyroptosis, or infections), resulting in AD-related neuroinflammation, neurotoxicity, and neurodegeneration

Neurodegeneration in ataxia telangiectasia (A-T) patients is caused by inheriting mutations in the *ATM* (A-T mutated) gene [81, 82], which encodes for the ATM protein kinase. Notably, a defect in the ATM-signaling is noted in the neuronal death in individuals with AD [81]. Accordingly, hemizygous *Atm*-deficient mice and certain AD mouse models exhibited a loss of ATM protein kinase functions in neurons [83]. Interestingly, the loss of neurons in the brain correlated with the AD disease stage [84]. Further, the loss of ATM protein kinase function in microglia resulted in accumulation of cytosolic DNA, which activates the ISD pathway [85]. As noted above, activation of ISD pathway in macrophages/microglia upregulates the Aim2 protein expression, activation of the Aim2 inflammasome, and pyroptosis. Therefore, the above observations are consistent with functional interactions between ATM protein kinase and Aim2 protein in modulating neuroinflammation and neurodegeneration (Fig. 3).

The ATM protein kinase family also includes DNA-dependent protein kinase (DNA-PK) [86]. Interestingly, binding of the Aim2 protein with the catalytic subunit of DNA-PK (DNA-PKc) inhibited its kinase activity and the DNA-PKc-mediated activation of the AKT protein kinase [87]. Further, a deficiency of the *Aim2* gene in epithelial cells [88] and hippocampus of the *Aim2*^−/−^.(B6.Sv129) mice [78] activated the AKT protein kinase. Of interest, a knockdown of the AIM2 protein expression in human normal lung fibroblasts (WI-38), as compared with control WI-38 cells, activated ATM protein kinase [62]. Similarly, macrophages from the *Aim2-*deficient mice, as compared with age- and sex-matched wild-type B6 mice, exhibited activation of the ATM protein kinase [51]. Because defects in the ATM kinase activation are associated with activation of the T1 IFN response [89] and activation of DNA-responsive inflammasome [90], studies are needed to assess the potential role of the Aim2/AIM2-ATM axis in modulating neuroinflammation and the development of AD.

## Conclusions

Suppression of T1 IFN response by Aim2 and AIM2 proteins in the CNS may be important to keep the levels of the T1 IFN-inducible negative regulator proteins (murine p202 and human IFI16-β and POP3) below a threshold to suppress neuroinflammation-related neurodegenerative diseases (Table 1). Notably, the generation of the *Aim2-*deficient 5XFAD AD mice on the pure B6 genetic background (*Aim2.*B6; 5XFAD) could provide novel insights into the precise role of the Aim2 protein in modulation of neuroinflammation and the development of AD. An improved understanding of the mechanisms by which Absent in Melanoma 2 proteins and their negative regulators modulate neuroinflammation could identify new approaches to treat AD.

**Table 1:** Murine Aim2 protein in neuroinflammation and neurodegeneration

Animal model(s)	Aim2 expression/function	Phenotype/effect	Ref.
ME7 prion, mouse	Aim2 expression upregulated	Neuroinflammation	[12]
*Aim2*^*−/−*^.B6.Sv129, mice	*Aim2* gene deficient	Lower locomotor activity, increased anxious behaviors, reduced fear memory	[46]
*Aim2*^*−/−*^.B6.Sv129, mice	*Aim2* gene deficient	Increased inflammation and adipogenesis; increased expression of the p202 protein	[59]
Traumatic brain injury, mouse	Aim2 inflammasome activation	Pyroptosis in brain microvascular endothelial cells, increased production of neuroinflammatory cytokines	[47]
Stroke, rat	Aim2 inflammasome activation	Production of neuroinflammatory cytokines	[45]
T9 spinal cord contusive injury, rat	Higher expression of Aim2 protein in neurons and microglia	Neuroinflammation	[48]
*Aim2*^*−/−*^*.*B6.Sv129; 5XFAD, mice	*Aim2* gene deficient	Reduced amyloid-β deposition in brain, increased expression of IL-6 and IL-18 cytokines in the brain	[23]
*Aim2*^*−/−*^*.*B6.Sv129; APP/PS1, mice	*Aim2* gene deficient	Increased spatial memory, altered structure of dendrites, increased dendritic spine densities	[78]

## Data Availability

Not applicable

## Abbreviations

AD: Alzheimer’s disease
AIM2: Absent in Melanoma 2
ATM: Ataxia telangiectasia
BBB: Blood-brain barrier
CARD: Caspase recruitment domain
cGAS: Cyclic GMP-AMP synthase
CNS: Central nervous system
DNA-PK: DNA-dependent protein kinase
IFN: Interferon
IFNAR: Interferon-α receptor
ISD: Interferon-stimulatory DNA
JAK: Janus kinase
IL-1: Interleukin 1
PBMCs: Peripheral blood mononuclear cells
POP3: Pyrin only protein
PYHIN: Pyrin and HIN domain containing interferon-inducible nuclear
STAT1: Signal transducer and activator of transcription-1
TBI: Traumatic brain injury
